# Priority setting to support a public health research agenda: a modified Delphi study with public health stakeholders in Germany

**DOI:** 10.1186/s12961-023-01039-w

**Published:** 2023-08-28

**Authors:** Dyon Hoekstra, Ansgar Gerhardus, Stefan K. Lhachimi

**Affiliations:** 1 Research Group for Evidence-Based Public Health, Leibniz-Institute for Prevention Research and Epidemiology (BIPS) & Institute for Public Health and Nursing Research (IPP), University of Bremen, Bremen, Germany; 2 Health Sciences Bremen, University of Bremen, Bremen, Germany; 3 Department of Special Needs Education and Rehabilitation, Carl Von Ossietzky University of Oldenburg, Oldenburg, Germany; 4 Department for Health Services Research, Institute for Public Health and Nursing Research (IPP), University Bremen, Bremen, Germany; 5 Department of Health, Nursing, Management, University of Applied Sciences Neubrandenburg, 17033 Neubrandenburg, Germany

**Keywords:** Priority setting, Health priorities, Stakeholder participation, Public health, Delphi technique

## Abstract

**Background:**

Research priority setting (RPS) studies are necessary to close the significant gap between the scientific evidence produced and the evidence stakeholders need. Their findings can make resource allocation in research more efficient. However, no general framework for conducting an RPS study among public health stakeholders exists. RPS studies in public health are rare and no such study has been previously conducted and published in Germany. Therefore, we aimed to investigate which research topics in public health are prioritised by relevant stakeholders in Germany.

**Methods:**

Our RPS study consisted of a scoping stage and a Delphi stage each split into two rounds. Firstly, we invited members of the German Public Health Association to gather expert insights during two initial workshops. Next, we defined the relevant stakeholder groups and recruited respondents. Thereafter, we collected research topics and assessment criteria with the respondents in the first Delphi round and aggregated the responses through content analysis. Finally, we asked the respondents to rate the research topics with the assessment criteria in the second Delphi round.

**Results:**

In total, 94 out of the 140 invited public health organisations nominated 230 respondents for the Delphi study of whom almost 90% participated in both Delphi rounds. We compiled a comprehensive list of 76 research topics that were rated and ranked by several assessment criteria. We split the research topics into two types, substantive research topics and methodological-theoretical research topics respectively, to ensure the comparability among the research topics. In both types of research topics—substantive research topics and methodological-theoretical research topics—the respective top five ranked research topics hardly differed between public health researchers and public health practitioners. However, clear differences exist in the priority ranking of many (non-top priority) research topics between the stakeholder groups.

**Conclusions:**

This research demonstrates that it is possible, with limited resources, to prioritise research topics for public health at the national level involving a wide range of pertinent stakeholders. The results can be used by research funding institutions to initiate calls for research projects with an increased relevance for health and/or scientific progress.

**Supplementary Information:**

The online version contains supplementary material available at 10.1186/s12961-023-01039-w.

## Background

The COVID-19 pandemic has made the importance of high-quality evidence abundantly clear to policy-makers. Hence, the pressure for policy-makers to gather and assess all available evidence when making decisions is increasing [1]. However, the process of including scientific evidence in public health decision-making has been—so far—not fully systematic and is complicated by barriers such as specific contexts and traditions, political priorities, individual beliefs and preferences, social values, and available resources [2–4].

Research also shows a significant gap between the scientific evidence that is produced by researchers and the actual scientific evidence demanded by policy-makers and other stakeholders [5–8]. Identifying what scientific evidence different groups of stakeholders prioritise may help to bridge this gap [7–9]. A promising yet underused approach is to involve stakeholders systematically in a structured priority setting process to ensure that their needs are accommodated by the produced evidence [10–14].

Funders of public health research have to decide which research projects to support while facing competing demands and scarce resources [15–18]. However, decision-making about which research should be conducted first is often not evidence-based [19]. Without rigorous research priority setting (RPS), funders risk that research topics will be chosen arbitrarily or are determined based on subjective goals [20–23]. Hence, RPS should be conducted in a structured matter to allow for better-informed decisions regarding the direction of future research investments.

### Research priority setting

Although several frameworks for priority setting in health research are suggested [14, 16, 17, 24–30], no established, single framework exists that fits all RPS purposes, not least due to varying aims and target groups in RPS [25, 31, 32]. Nevertheless, several studies on health-related RPS agree that a best-practice RPS is a multi-stage process combining multiple methodological approaches [15, 29, 31].

Most notably, many studies incorporated Delphi-like techniques in RPS [33]. The Delphi technique often consists of two or more rounds, in which a panel of experts can give their opinions about an issue. In the following Delphi rounds, they are encouraged to give anonymous controlled feedback to the results of previous stages, which allows them to reflect, reassess and revise their opinions and judgements if needed [34–36]. The Delphi technique in health research is especially appropriate when seeking shared preferences from multiple stakeholders and when available evidence is incomplete [34, 35, 37, 38]. The implementation of this technique assumes that a result yielded from a larger panel of experts with various views will be more well-grounded than a conclusion reached by only one stakeholder [34]. Furthermore, the Delphi technique offers additional advantages, such as uncomplicated incorporating various metrics-based techniques and methods for consensus building [35, 38]. The Delphi technique also does not require face-to-face interactions, which reduces time and cost investments. More importantly, this reduces potential drawbacks of the in-person approach that lead to inaccurate estimations because of social pressure and/or dominance of individuals within groups [31, 39, 40].

Moreover, rating of research topics should preferably be done using more than a single assessment criterion to measure different dimensions of why specific topics are prioritised [23, 29, 31].

RPS studies focussing on the field of public health are rare: Selected public health topics are considered occasionally in RPS studies that focus on health in general or on a (sub)field that overlaps with public health (e.g. health services [41], nursing [42], obesity [43] or mental health [44]). We identified three RPS studies [40, 45, 46] that focused specifically on all public health topics, but for the purpose of prioritising topics for conducting systematic literature reviews for the Cochrane Collaboration only.

In Germany, where public institutions fund more than 3 billion EUR per year on health research [47], no RPS study specifically encompassing all public health research topics has been published so far.

### Objectives

Our main objective was to investigate which research topics in public health should be prioritised in Germany according to a wide range of stakeholders by conducting a structured priority setting study. We also wanted to identify potential similarities and differences between stakeholder groups.

## Methods

**Fig. 1:**
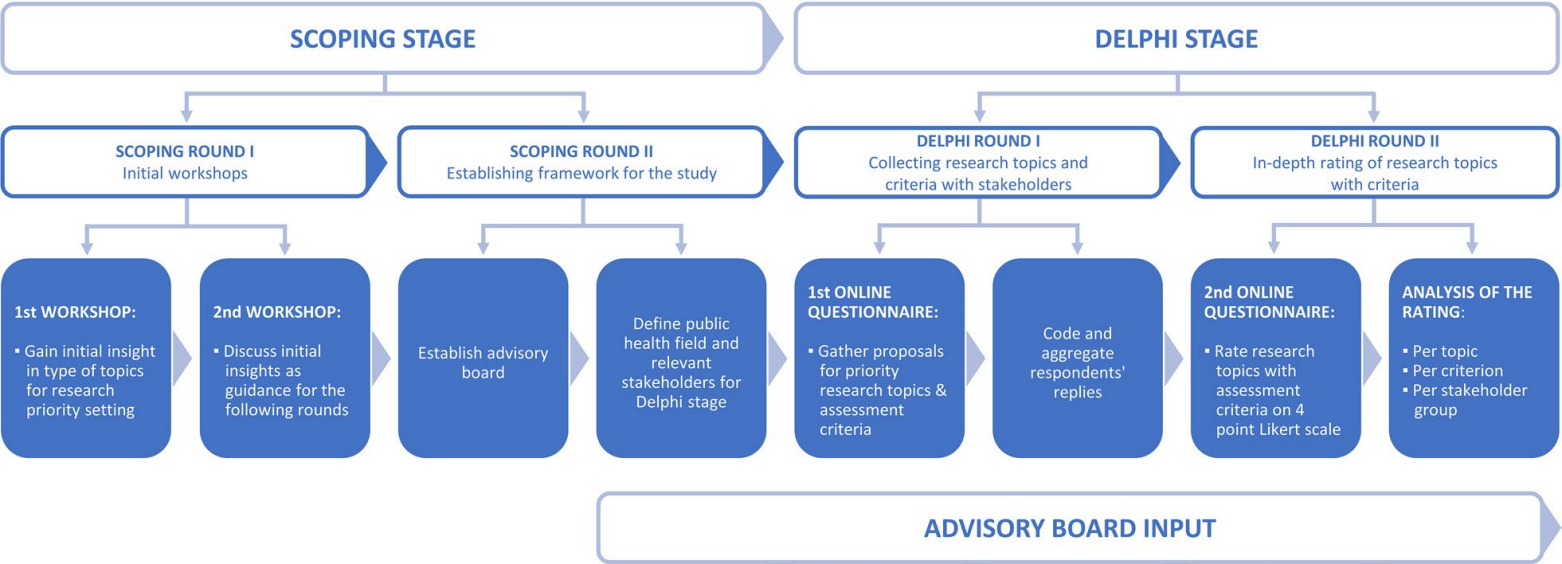
The prioritisation process

Our RPS study (see Fig. 1) was conceptually split into a scoping stage and a Delphi stage and consisted of four rounds:

- Scoping round I. Gathering expert insights during two initial workshops
- Scoping round II. Establishing the framework for the study
- Delphi round I. Collecting research topics and assessment criteria with stakeholders
- Delphi round II. Rating the collected research topics with the collected assessment criteria

In the scoping stage, we conducted two initial workshops, established an advisory board, and set the framework for the remainder of the study. For the Delphi stage, we administered two rounds of online questionnaires.

### Scoping round I—initial workshops

We conducted our study in collaboration with the German Public Health Association (DGPH). Participants of two workshops, organised by the DGPH in 2015 and 2016, discussed and proposed potential research topics that should be covered in an RPS study. The invitations for the workshops were sent to all members of the DGPH. Additionally, individuals with a clear public health expertise, mainly consisting of established researchers in Germany and representatives of German research funding agencies, were invited. In total, the number of participants during the workshops varied between 40 and 50 individuals.

During the first workshop called “Priority topics for public health research”, members of the DGPH were invited during a 5-h-long exploratory roundtable discussion to discuss which research topics for public health research need most attention. The workshop was publicly announced and open to all members of the DGPH. The participants could propose topics or broader research areas themselves for different domains of public health and they could critically reflect on the topics that were proposed by other participants. The exploratory results were recorded in the minutes and distributed to the members of the DGPH by email.

In order to frame the discussions in this stage regarding potential research topics, we defined domains, i.e., broader public health research areas, to group similar research topics together to ensure comparability*.* We also used the domains as guidance for the respondents in the first Delphi round, allowing the respondents to consider the different broader public health research areas. The domains were

- Research on current/contemporary issues
- Effectiveness research
- Policy research
- Implementation and/or participatory research
- Theories and theoretical concepts
- Methodological research
- Research on indicators

The second 2-h-long workshop took place during the annual conference of the German Society for Social Medicine and Prevention (DGSMP). In this plenary discussion the results of the first workshop and the planning for our RPS study were presented and discussed with representatives of German research funding agencies.

### Scoping round II—establishing framework for the study

The author team approached established researchers and practitioners in the field of public health in Germany to form an advisory board. The advisory board consisted of five researchers and practitioners in total (see also the acknowledgements below), with both subject matter and methodological expertise [25, 31, 48].The advisory board members were not allowed to participate in the following Delphi stage.

The advisory board members reviewed the design and the proposed analyses of the study, particularly with regards to the scoping stage of the study and the questionnaire design. The advisory board was in particular helpful for framing if a research topic can be reasonably considered within the realm of public health research. In our definition for this study, public health research encompasses population level and health systems research, which excludes research topics that are predominantly clinical or biomedical research.

Moreover, we identified the stakeholder groups that are active in German public health and who are therefore relevant for inclusion in our RPS study [25, 44, 49]:

- Public health research and education
- Public health administration and policy-making
- Non-governmental organisations (NGO) and representatives of the public
- Representatives of health professionals and health care institutions
- Self-governing associations of health providers and statutory health insurance

The stakeholder groups stand for different professional fields who are either producers, facilitators, or consumers of public health research in Germany. Hereafter, we identified specific organisations that fall within each of the stakeholder groups. We used this list of organisations for the recruitment of individual respondents for the Delphi stage (see Additional file 1 for a full list).

### Delphi round I—collecting research topics and assessment criteria

In the first Delphi round, we distributed an online questionnaire to individual respondents. We presented the initial list of proposed research topics from the workshops and proposed assessment criteria that were defined during the scoping stage in the online questionnaire. The respondents could vote from this list which research topics should be included and which assessment criteria should be used for the rating of the research topics in the second Delphi round. Moreover, the respondents could propose further research topics and assessment criteria.

Thereafter, we coded and aggregated all proposed research topics using content analysis [50–52] to minimize redundancies and overlaps. The aggregated research topics should be interpreted as broader research themes that can be used for, e.g., setting the focus of future calls for proposals.

### Delphi round II—in-depth rating of research topics with assessment criteria

In the second Delphi round, we presented the final list of research topics based on the first Delphi round. For each research topic, we also reported how many respondents voted for a particular research topic during the previous stage [15, 29, 53]. Subsequently, the respondents were asked to rate each research topic with the assessment criteria on a 4-point Likert scale (also with the alternative choice “I cannot assess this"). In order to limit the workload for the respondents, we presented each respondent a random sample of approximately 50% of the research topics only.

### Recruitment

We based our recruitment strategy of respondents for the Delphi stage on a previous priority setting study in the field of health services research [41]: We invited public health relevant organisations, i.e. stakeholders, to nominate individuals as respondents from within their own organisation for participation in the study. We asked the organisations to nominate individuals who they believed are most suitable for assessing and prioritising research topics in public health. We pre-defined this as somebody who had multiple years of experience working in the field of public health and who is either a researcher or a user of research.

In total, we invited 140 organisations to nominate up to three respondents each. Table 1 shows the number of organisations by stakeholder group (see Additional file 1 for a complete list of the invited organisations).

**Table 1:** Respondents during the Delphi stage by stakeholder group

Stakeholder group	Invited organisations to nominate respondents for the Delphi stage	Organisations that nominated respondents for the Delphi stage	Participation 1st questionnaire (Delphi round I)	Participation 2nd questionnaire (Delphi round II)
	Nr. of organisations	Nr. of organisations	Nr. of respondents	Nr. of respondents
Research and/or higher education	61 (44%)	47 (50%)	105 (52%)	98 (48%)
Administration and/or politics	35 (25%)	20 (21%)	24 (12%)	32 (16%)
Representatives of the general public	22 (16%)	11 (12%)	37 (18%)	39 (19%)
Healthcare professionals	13 (9%)	10 (11%)	8 (4%)	7 (3%)
Statutory health insurance	9 (6%)	6 (6%)	11 (5%)	13 (6%)
None of the above	n.a	n.a	4 (2%)	2 (1%)
Missing	n.a	n.a	12 (6%)	12 (6%)
Total	140 (100%)	94 (100%)	201 (100%)	203 (100%)

The first two columns of Table 1 show the number of invited organisations by stakeholder group and how many actually nominated respondents. The last two columns show how many of these nominees participated in Delphi round I and how many in Delphi round II

Of the 140 invited organisations, 94 (67%) participated and nominated in total 230 individuals. The participation rate in the first Delphi round was 87% (201 respondents) and in the second Delphi round 88% (203 respondents).

Table 1 also shows the professional area the respondents assigned themselves: approx. 50% of the respondents are working in research and/or higher education. The other respondents assigned themselves to the following stakeholder groups: Administration and/or politics, representatives of the general public, self-governing associations of health providers and statutory health insurance, and healthcare professionals. The respondents had an average of 16 years of experience in the field of public health.

## Results

We will report the results of each Delphi round in turn.

### First Delphi round: collecting research topics and assessment criteria with stakeholders

The respondents voted for research topics and assessment criteria from the initial expert list and could also propose further research topics and assessment criteria for inclusion in the second Delphi round. In total, the respondents proposed 529 research topics and 50 assessment criteria which we aggregated through a content analysis into 76 sufficiently distinct research topics and 6 assessment criteria, respectively.

The content analysis of the assessment criteria revealed, however, two types of research topics that could not be assessed meaningfully with the same set of criteria: Substantive research topics and methodological-theoretical research topics. Substantive research topics focused on specific thematic contents, for example “Climate change and health” or “Health literacy”. Methodological-theoretical research topics focused on the application or development of specific research methods and paradigms as well as on the development of theories and concepts for public health research, for example “Participation in health research” or “Interdisciplinary research”. This split resulted in 46 substantive research topics and 30 methodological-theoretical research topics, each with three associated assessment criteria. The operational definitions of the 6 assessment criteria are shown in Box 1 (see Additional file 2a and b for the final list of research topics based on the content analysis).

Box 1: List of assessment criteria for substantive (S1 to S3) and methodological-theoretical (M1 to M3) research topics

**Table:** 

Assessment criteria	Operational definition
S1 Improving health	Research on the topic can contribute to a substantial improvement of general health (e.g. reducing the burden of disease, promoting physical & mental health)
S2 Health justice	Research on the topic can contribute to greater health justice (e.g. increasing health equity and equality)
S3 Insufficient research	Research results on the topic are insufficient (e.g. do not exist, are of inadequate quality, are not up-to-date, or will gain importance in the future)
M1 Impact on public health research	Methodological and/or theoretical research on the topic can have a substantial effect on further public health research
M2 Impact on public health practice	Methodological and/or theoretical research on the topic can substantially improve policy and practice
M3 Potential for innovative insights	Methodological and/or theoretical research on the topic has a high potential for innovative findings

### Second Delphi round: rating of research topics

In the second Delphi round the respondents rated the research topics using the assessment criteria on a 4-point Likert scale, ranging from 1 “totally agree” to 4 “totally disagree”. Alternatively, the respondents could also select the option “I cannot assess this”.

Table 2 shows the average rating by assessment criterion. Methodological-theoretical research topics received on average lower ratings than substantive research topics. Furthermore, for the two criteria that required broader insight into existing public health research (criterion S3 “Insufficient research” and criterion M3 “Potential for innovative insights”), the share of respondents who answered “I cannot assess this” is highest.

**Table 2:** Assessment results per criterion of both substantive and methodological-theoretical research topics

Substantive research topics (*n* = 46)				Methodological-theoretical research topics (*n* = 30)			
	Average rating score of all topics	Average nr. of respondents per topic	Share of assessments “I cannot assess this”		Average rating score of all topics	Average nr. of respondents per topic	Share of assessments “I cannot assess this”
Criterion S1: Improving health	1.87 *95% CI: [1.85; 1.90]*	81	4%	Criterion M1: Impact on public health research	2.04 *95% CI: [2.00; 2.07]*	77	4%
Criterion S2: Health justice	1.95 *95% CI: [1.92; 1.98]*	80	5%	Criterion M2: Impact on public health practice	2.07 *95% CI: [2.03; 2.10]*	76	5%
Criterion S3: Insufficient research	2.05 *95% CI: [2.02; 2.08]*	73	12%	Criterion M3: Potential for innovative insights	2.10 *95% CI: [2.06; 2.13]*	73	9%

This table shows the results of the assessments of all the respondents per criterion. The respondents had to rate the research topics according to the assessment criteria on a 4-point Likert scale, ranging from 1 “totally agree” to 4 “totally disagree”. Alternatively, the respondents could also select the option “I cannot assess this”.

We asked each respondent to assess approximately 50% of all the research topics in order to reduce the workload. The selection and order of the research topics that each respondent had to rate was randomised. Therefore, the total number of respondents that assessed a certain topic differs slightly.

### Substantive research topics

As shown in Table 3, the average overall rating score for all substantive research topics was 1.96, ranging from 1.42 for the research topic “Interventions in everyday life” (ranked highest) to 2.48 for the research topic “Accidents, violence, self-harm” (ranked lowest).

**Table 3:** Results of the rating of substantive research topics

Research topic	Overall			S1 Improving health			S2 Health justice			S3 Insufficient research		
	Score	Rank	Not assessed (in %)	Score	Rank	Not assessed (in %)	Score	Rank	Not assessed (in %)	Score	Rank	Not assessed (in %)
Interventions in everyday life	1.42	1	2	1.34	1	0	1.38	2	1	1.55	2	4
Health in all policies	1.57	2	7	1.52	6	6	1.53	3	4	1.66	6	11
Social inequality and injustice	1.58	3	3	1.40	2	2	1.36	1	2	1.99	20	4
Impact of health policy measures	1.60	4	7	1.57	9	2	1.67	8	5	1.57	3	14
Community-based prevention and health promotion	1.61	5	4	1.56	8	3	1.57	5	1	1.69	7	8
Implementation Research	1.61	6	8	1.51	5	5	1.73	12	6	1.60	5	13
Intervention studies over longer periods of time	1.68	7	3	1.49	4	0	1.98	28	5	1.59	4	4
Improving health care	1.71	8	4	1.53	7	3	1.63	7	0	1.96	16	8
Effectiveness of municipal / community-oriented approaches	1.72	9	8	1.69	12	4	1.76	13	3	1.70	8	16
Health literacy promotion	1.73	10	4	1.69	14	2	1.62	6	3	1.87	12	9
Health and children/youth/family	1.78	11	4	1.42	3	1	1.56	4	4	2.37	37	8
Digitisation and health	1.80	12	5	1.72	16	3	2.14	35	3	1.54	1	10
Interdependencies between society, setting and individual health	1.80	13	3	1.84	22	1	1.72	11	1	1.84	10	5
Health and ageing	1.81	14	1	1.69	13	0	1.69	9	1	2.04	23	3
Knowledge translation	1.84	15	8	1.76	17	3	1.80	15	6	1.97	17	16
Research with focus on specific target groups	1.84	16	6	1.70	15	3	1.71	10	5	2.13	29	9
Global Health and effects of globalisation	1.86	17	10	1.86	25	5	1.86	19	5	1.85	11	18
Environment/climate change and health	1.87	18	7	1.83	20	3	1.89	21	4	1.90	14	13
Patient and user orientation	1.89	19	6	1.82	19	3	1.98	27	4	1.88	13	12
Health Communication	1.90	20	5	1.83	21	1	1.83	17	3	2.05	24	10
Influence through economisation and interest groups	1.91	21	9	2.07	33	9	1.91	22	9	1.73	9	11
Prevention of non-communicable diseases	1.92	22	6	1.68	11	3	1.99	29	3	2.10	25	12
Research on health care needs	1.96	23	5	1.86	24	3	1.80	14	0	2.23	32	11
Life course perspective	1.97	24	8	1.95	27	2	1.99	30	8	1.98	19	12
Work and health	1.97	25	2	1.67	10	2	1.85	18	1	2.41	42	3
Migration health	1.98	26	6	2.10	36	3	1.87	20	5	1.97	18	9
Mental health	1.99	27	7	1.77	18	5	1.94	24	5	2.24	34	9
Governance (global, national, regional) and health systems	2.00	28	11	2.03	30	5	1.96	25	5	2.03	22	22
Nutrition and health (cultural, physiological, social)	2.01	29	4	1.84	23	1	1.81	16	6	2.37	38	5
Health policy analysis	2.03	30	6	2.12	37	4	1.97	26	5	2.00	21	8
Disability and multimorbidity	2.04	31	9	1.96	28	5	1.93	23	5	2.22	31	19
Sustainability	2.09	32	9	1.99	29	8	2.32	40	7	1.95	15	12
Diversity and gender	2.13	33	6	2.13	38	2	2.03	31	4	2.23	33	13
Resilience	2.14	34	7	2.10	35	5	2.20	36	4	2.12	28	13
Sustainable Development Goals (SDGs)	2.14	35	19	2.21	40	20	2.12	33	13	2.10	26	24
Behaviour change measures	2.17	36	4	2.04	31	1	2.14	34	3	2.34	36	7
Research on health professions	2.22	37	6	2.07	32	4	2.43	44	2	2.15	30	14
Infectious diseases and vaccination protection	2.23	38	5	1.93	26	2	2.20	37	1	2.55	46	11
One Health	2.23	39	25	2.27	41	19	2.31	39	21	2.12	27	34
Health reporting	2.24	40	5	2.09	34	3	2.10	32	1	2.54	45	11
Self-help	2.30	41	7	2.28	42	6	2.25	38	1	2.39	41	13
Effectiveness of counselling at the individual level	2.33	42	5	2.37	44	1	2.37	41	3	2.25	35	13
Population perspective on pharmaceuticals	2.34	43	14	2.17	39	10	2.49	45	6	2.37	39	24
Health economic evaluation	2.37	44	8	2.31	43	4	2.43	43	9	2.37	40	11
Public health crises and disasters	2.41	45	12	2.38	45	9	2.42	42	8	2.45	43	19
Accidents, violence, self-harm	2.48	46	12	2.39	46	7	2.54	46	10	2.51	44	20
Average	1.96		7	1.87		4	1.95		5	*2.05*		12

In addition to the rating score, we also provided the rank of each research topic. For some research topics, the ranking per criterion showed slight differences, whereas for other research topics we found larger differences. For example, the research topic “Social inequality and injustice” was not ranked very high according the assessment criterion “Insufficient research” (ranked 20th out of 46); however, according to the assessment criteria “Improving health” and “Health justice”, the same research topic was ranked very high (respectively, 2nd and 1st).

### Methodological-theoretical research topics

Table 4 shows the rating and ranking of methodological-theoretical research topics for the three assessment criteria separately (criteria M1 to M3). The average overall rating score for all methodological-theoretical research topics was 2.07, ranging from 1.64 for the research topic “Interdisciplinary research” (ranked highest) to 2.46 for the research topic “Mobility concepts” (ranked lowest). For all the three criteria, the highest-ranked research topics were virtually identical. For other (not highly-ranked) research topics we found larger ranking differences between the criteria. For example, the research topic “Comparative effectiveness research (CER)” was ranked 9^th^ with the assessment criterion “Potential for innovative insights”. With the assessment criteria “Impact on public health research” and “Impact on public health practice”, the same research topic was ranked much lower (17th and 25th, respectively).

**Table 4:** Results of the rating of methodological-theoretical research topics

Research topic	Overall			M1 - Impact on public health research			M2 - Impact on public health practice			M3 - Potential for innovative insights		
	Score	Rank	Not assessed (in %)	Score	Rank	Not assessed (in %)	Score	Rank	Not assessed (in %)	Score	Rank	Not assessed (in %)
Interdisciplinary research	1.64	1	2	1.68	3	2	1.64	1	0	1.61	1	4
Complex interventions	1.68	2	5	1.67	2	1	1.71	2	7	1.67	2	9
Evidence-based public health research	1.76	3	3	1.60	1	4	1.81	5	1	1.87	4	5
Further development of intervention studies	1.81	4	4	1.77	6	4	1.78	4	3	1.87	5	7
Transdisciplinary research	1.83	5	5	1.76	5	5	2.06	14	3	1.67	3	8
Participation in health research	1.85	6	4	1.81	7	2	1.82	6	1	1.91	7	8
Indicators for the quality of health care and public health	1.86	7	5	1.74	4	1	1.77	3	6	2.06	12	6
Population participation	1.94	8	2	1.94	11	1	1.95	10	1	1.93	8	4
Development and maintenance of good health	2.00	9	4	2.04	13	3	1.87	7	5	2.10	13	4
Systematic reviews	2.03	10	4	1.91	8	4	1.96	11	1	2.21	21	7
Indicators of health literacy	2.04	11	5	2.18	19	5	1.90	8	2	2.04	10	6
Internationally comparable indicators	2.06	12	5	1.93	10	4	1.99	13	4	2.27	25	7
Structural and process indicators	2.06	13	5	2.10	15	4	1.98	12	4	2.11	15	8
Causal analyses / experiments	2.07	14	3	2.11	16	3	2.20	21	2	1.89	6	2
Qualitative health studies	2.08	15	2	1.92	9	2	2.15	19	0	2.16	18	4
Indicators of quality of life and of positive health states	2.09	16	4	2.23	20	3	1.91	9	5	2.14	16	5
Process evaluation	2.12	17	5	2.07	14	2	2.07	15	6	2.21	20	7
Methodological research on registry and routine data	2.12	18	4	2.04	12	3	2.13	18	3	2.19	19	6
Big Data	2.14	19	10	2.16	18	10	2.23	22	8	2.04	11	13
Comparative Effectiveness Research (CER)	2.16	20	15	2.15	17	9	2.30	25	12	2.02	9	24
Sociological aspects of health	2.16	21	4	2.23	21	1	2.15	20	1	2.11	14	10
Conceptualization of behaviour/setting	2.16	22	7	2.26	23	5	2.08	17	4	2.16	17	11
Indicators for health targets	2.28	23	5	2.26	22	4	2.07	16	5	2.51	30	5
Modelling Studies—Decision Analysis	2.29	24	13	2.30	25	8	2.29	24	12	2.29	26	18
Theoretical foundation of effects models	2.31	25	6	2.35	26	5	2.34	26	4	2.24	23	9
Action Research	2.31	26	13	2.42	28	10	2.29	23	8	2.22	22	20
Research on public health theories	2.33	27	6	2.27	24	7	2.37	27	3	2.35	28	10
Preventive markers	2.41	28	9	2.48	29	5	2.40	28	5	2.35	27	17
Online Social Research	2.44	29	12	2.65	30	5	2.41	29	15	2.26	24	17
Mobility concepts	2.46	30	12	2.37	27	9	2.56	30	8	2.45	29	20
Average	2.07		7	2.04		4	2.07		5	2.10		9

### Comparison by stakeholder groups

As shown in Table 1, approximately 50 percent of the respondents belonged to the *"*Public health research and/or higher education*"* group. The other stakeholder groups, *"*Representatives of the general public*"*, *"*Administration and/or politics*"*, *"S*elf-governing associations of health providers and statutory health insurance*"* and *"*Healthcare professionals*"* were grouped together and defined as *"*Public health practitioners*"*. This allowed for a comparison between these two major stakeholder groups.

Additional file 3a and b show the ranking of substantive and methodological-theoretical research topics, respectively, by stakeholder group. For the majority of the substantive research topics, we did not see a major difference between the two stakeholder groups in the ranking. For nine research topics, however, there was a very large difference of ten or more positions in the ranking: The research topics "Digitalisation and health", "Nutrition and health (cultural, physiological, social)", and "Infectious diseases and vaccination" showed the largest differences (all ranked 14 places higher by public health practitioners as compared to public health researchers). Among the methodological-theoretical research topics (see Additional file 3b), we saw a very large difference (ten or more positions) in the ranking of seven methodological-theoretical research topics, with the research topics "Process evaluation", "Causal analyses / Experiments", and "Modelling studies / Decision analysis" showing the largest difference (up to 15 ranking positions difference).

As the judging difference in ranking as *"*large*"* or *"*very large*"* may seem somewhat arbitrary, we calculated the interrater reliability by stakeholder group, i.e., public health researchers vs. public health practitioners. The interrater reliability can be quantified by calculating an agreement coefficient (AC). We calculated Gwet’s AC because it allows for multiple rates and multiple topics [54, 55]. The interpretation is straightforward: Values close to 1 indicate almost total agreement among the respondents, values close to 0 indicate agreement is mostly due to chance. The analysis was conducted using the Stata software version 17 [56, 57].

In Table 5 we report the degree of agreement among respondents by the two major stakeholder groups (public health researchers vs. public health practitioners). For all three substantive assessment criteria the agreement of raters within both stakeholder groups could be considered as moderate (Gwets AC between 0.41 and 0.60). The agreement was highest for the criterion S1 (“Improving health”). For the criterion S3 (“Insufficient research”), the agreement among public health researchers and public health practitioners was the lowest among the substantive assessment criteria and the difference between both stakeholder groups was not statistically significant anymore.

**Table 5:** Agreement coefficient† by stakeholder groups (public health researchers vs. public health practitioners) for each criterion

Criterion	PH researchers		PH practitioners		*T*-test††
	Gwets AC†	S.E	Gwets AC†	S.E	*p*-value
S – all criteria	0.56	0.01	0.50	0.01	0.001
S1 (improving health)	0.60	0.02	0.54	0.03	0.01
S2 (health justice)	0.57	0.02	0.49	0.02	0.002
S3 (insufficient research)	0.53	0.02	0.48	0.02	0.08
M – all criteria	0.47	0.02	0.35	0.02	0.001
M1 (impact on PH research)	0.48	0.03	0.32	0.04	0.001
M2 (impact on PH practice)	0.41	0.04	0.35	0.03	0.15
M3 (potential for innovative insight)	0.52	0.02	0.39	0.02	0.001

Agreement coefficient†, i.e., degree of agreement among raters by stakeholder groups (public health researches vs. public health practitioners) for each criterion and *t*-test whether the difference between stakeholder groups is statistically significant. Criteria S1 to S3 are the assessment criteria for the substantive research topics. Criteria M1 to M3 are the assessment criteria for methodological-theoretical research topics

†Interpretation of coefficient: 0.0–0.20 = slight agreement; 0.21–0.40 = fair agreement; 0.41–0.60 = moderate agreement; 0.61–0.80 = substantial agreement; 0.81–1 = almost perfect agreement

††T-test to investigate the difference between stakeholder groups

For all three methodological rating criteria the agreement among public health researchers could be considered moderate (Gwets AC between 0.41 and 0.60). However, among public health practitioners there was only fair agreement (Gwets AC between 0.21 and 0.40). Moreover, the coefficients for the methodological-theoretical assessment criteria were slightly lower than for the substantive assessment criteria. Concerning criteria M2 (“Impact on public health practice”) there was no statistically significant difference between the two groups of stakeholders.

## Discussion

Funding decisions in public health research are complex and multiple criteria play a role. Consequently, priority setting becomes a challenge that policy-makers cannot solve easily [23, 58]. Therefore, policy-makers need structured and transparent RPSs that take all relevant criteria into account [25, 29, 59].

We conducted an RPS study with the aim to investigate which research topics in public health should be prioritised in Germany according to different stakeholders. To the best of our knowledge, we conducted the first structured and transparent RPS study for public health research topics in Germany.

Improved transparency in RPS can strengthen the acceptability of the prioritised research topics, not least because research efforts and funding can be directed towards research that is relevant to all stakeholders [14]. Involving public health researchers and policy-makers simultaneously in RPS studies is not rare, however, Cortier and colleagues [60] demonstrated that other stakeholder groups such as public and advocacy organisations are not frequently included. By using a modified Delphi technique, we involved a wide range of stakeholders in order to investigate which research topics should be prioritised in public health in Germany.

We used a multi-stage approach to balance two conflicting aims, i.e., (i) eliciting proposals from a wide range of stakeholders and (ii) yet ensure that the proposals are within the realm of public health research. In order to ensure the latter, we defined domains that each stand for a broader public health research area and created—by conducting expert workshops in cooperation with the DGPH—an initial list of relevant research topics.

In the first Delphi round, we collected additional proposals for research topics from the participating stakeholders. The findings of the first Delphi round demonstrated clearly the need to split research topics into two groups (46 substantive vs. 30 methodological-theoretical research topics). We applied a different set of assessment criteria to these two types of research topics in the second Delphi round, as methodological-theoretical research topic cannot be assessed by the same criteria as substantive research topics.

The overall rating score for a particular research topic is the average of the rating scores for the three corresponding assessment criteria. No research topic received an overall score lower than 2.5. That is, on average no research topic was considered unimportant.

We found large differences in the rating and ranking of the research topics when differentiating the results along the three assessment criteria. These results corresponded to our expectation that the assessment of a particular research topic depends on the criterion applied (e.g. improving population health vs. insufficient research). It shows that the assessment criteria are measuring distinct dimensions of a research topics and can give an indication on why a particular research topic is prioritised high or low. Although many studies highlighted the importance of selecting multiple assessment criteria that fit to the specific context and that can sufficiently discriminate between the assessment of different research topics [25, 29, 31, 61], most RPS studies do not involve stakeholders in the selection of relevant assessment criteria [60]. The use of multiple assessment criteria also makes it easier for the respondents to rate the research topics as it provides clarity of what aspect of the research topic they were rating exactly.

The descriptive comparison of the priority ranking of the research topics by stakeholder groups, showed that both public health practitioners and public health researchers ranked predominantly similar research topics as top priorities. However, it should also be noted that clear differences exist in the priority ranking of many (non-top priority) research topics between the two stakeholder groups. Moreover, the degree of agreement among the respondents on the importance of research topics differed by stakeholder group: Public health practitioners had on all criteria a lower degree of agreement than public health researchers. This overall lower degree of agreement among public health practitioners might be a result from the existing variation within the stakeholder group, as several more narrowly defined stakeholder groups were aggregated together and labelled ‘public health practitioners´. Further research is needed to investigate how different and/or more narrowly defined stakeholder groups might produce differing results in an RPS. However, the group sizes of the more narrowly defined stakeholder groups in our RPS study were too small, which prevented a comparison between them.

Public health is a complex and multifaceted field that is mainly conducted in a real-life setting, which makes it a challenge for researchers and other stakeholders in public health research to develop a common understanding of what research is most relevant or important [62].The top priorities derived from our study (see Tables 3 and 4) also clearly highlight the importance of inter- or transdisciplinary research in a complex and real-life setting.

Funders in Germany can use the results of this RPS study to discuss future calls in a transparent and structured manner. The overall results show which research topics are prioritised highest by a respective stakeholder groups, while the assessment criteria also help to explain why particular topics are rated lower or higher. A worthwhile next research step should be to investigate if and how funders and other policy-makers in Germany make use of the findings of this RPS study.

### Strengths and limitations

This is, to our knowledge, the first RPS study for public health research in Germany. So far no widely accepted standards exist on how to conduct such an RPS. Hence, our works was in many ways novel, but limitations have to be acknowledged.

We recruited respondents for the Delphi stage by identifying relevant organisations in the field of public health and ask them for the nomination of respondents. The approach may seem onerous, but it has been successfully applied already before [41] and had several advantages: The workload and cost for recruiting was relatively low and the approached organisations provided the contact details of the respondents. Furthermore, we had a very high participation rate (close to 90%) in both Delphi rounds as compared to other RPS studies that did not use this specific recruitment approach for their Delphi stage [13, 63, 64]. Also, the approached organisations nominated respondents they considered to have sufficient expertise in public health to participate meaningfully in an RPS study.

The validity of the sample is limited by the specific public health organisations we identified in our search (see Additional file 1) that nominated respondents. As organisations could only nominate up to three respondents, no single organisation could influence the rating unduly. We explicitly refrained from asking the organisations to form and present an official position; we merely asked their nominees to propose and prioritise research topics based on their expertise. In absence of a readily available list of all relevant public health experts and/or stakeholders in Germany, the only alternative would have been an open call to participate in the Delphi stage with all its disadvantages (e.g., unclear participation rates, unclear biases, and uncertain expertise of respondents).

Furthermore, we did not collect data on the context of the respondents within their organisations, such as position, decision-making power and organizational culture, although these factors could have an influence on the actual responses. However, we believe that this was necessary in order to ensure the anonymity of the respondents and to limit the time respondents had to spend for participating in our modified Delphi, therefore ensuring a sufficiently high participation rate. Future research is needed to investigate how contextual factors influence the RPS.

Our RPS approach incorporated a modified Delphi technique, i.e., the results of the rating in the second Delphi round were not discussed by the respondents in an additional follow-up round. We modified the Delphi process to reduce the workload for the respondents and to be able to include more stakeholders. Therefore, the results of our Delphi study should be interpreted as an indication of preferences rather than a consensus. On the other hand, this modified process increased the involvement of stakeholders who otherwise might be marginalised in the discussion of a full Delphi study, or who would not be invited at all as one of the top experts for a Delphi study [35, 65]. The open nature of the first Delphi round, i.e., the possibility to propose additional research topics, required us to conduct a content analysis to aggregate the large number of redundant or overlapping proposals (529 research topics) into a practicable number of sufficiently distinct research topics. However, an aggregation of data often means a loss of information. Furthermore, we are also aware that the formulation of the research topics through the content analysis is based on partly subjective assessments by the author team; a procedure that is however inherent to content analysis and a well-established method for condensing vast amounts of qualitative data [51, 52, 66–68].

Despite wide agreement that distinct criteria should be applied to rate the different dimensions of a research topic, no standard exists. We used the input of the respondents and were able to develop and apply 6 distinctive assessment criteria. However, further research is needed to investigate if additional or perhaps different assessment criteria are needed. Moreover, it is not clear if generic assessment criteria exist that can be used in any RPS study or if they must be specific to the research field, e.g., public health vs. health service research. Nevertheless, our study demonstrated that for public health research topics, depending on whether a research topic is substantive or methodological-theoretical, different assessment criteria should be used.

As this is the first RPS study of its kind in Germany that considers all public health research domains, it is unclear how long the results of the study remain sufficiently valid to inform policy-makers. This study was conducted before the outbreak of the Covid-19 epidemic in 2020 and an update may already yield different results. However, we do believe that many proposed research topics, such as “Health in all policies” or “Health literacy promotion”, will be considered relevant for the foreseeable future. A replication of the study after a few years would help to distinguish which research topics are rather long-term priorities and which research topics are rather short-term.

Funders and other research teams could easily use our study as a template for their own RPS studies in a different field of study. Although conducting our structured RPS study is a time intensive exercise initially, we believe that a follow-up study would not need much additional work.

## Conclusions

We conducted a structured RPS study involving a wide range of stakeholders in the field of public health in Germany. Our study demonstrates how a multi-stage RPS study, using an anonymous and online modified Delphi technique in which stakeholders rate and rank a comprehensive list of research topics, can be implemented with limited resources. Being the first structured RPS study for public health that can be replicated easily, it may lay important groundwork for future RPS studies in public health and related fields of health research.

### Supplementary Information

**Additional file 1.** List of organisations that were invited to participate in the study—by stakeholder group. We identified specific organisations that fall within each of the included stakeholder groups. The stakeholder groups stand for different professional fields who are either producers, facilitators, or consumers of public health research in Germany. We used this list of organisations for the recruitment of individual respondents for the Delphi stage.

**Additional file 2.** Final list of research topics after content analysis—Incl. how many respondents´ suggestions were aggregated into the research topic. This file represents the results of the content analysis after the first Delphi round. In total, the respondents proposed 529 research topics and 50 assessment criteria in the first Delphi round, which we aggregated through a content analysis into 76 sufficiently distinct research topics and 6 assessment criteria, respectively. a and 2b show the final list of research topics based on the content analysis for the substantive and the methodological-theoretical research topics, respectively.

**Additional file 3.** Comparison of the ranking of research topics between public health researchers versus public health practitioners. a and b show the comparison of the ranking of substantive and methodological-theoretical research topics, respectively, by stakeholder group (public health researchers versus public health practitioners). A difference in minus means the research topic is ranked higher by public health researchers; a difference in plus means the research topic is ranked higher by public health practitioners.

## Data Availability

Available upon request.

## Abbreviations

RPS: Research priority setting
DGPH: German Public Health Association (Deutsche Gesellschaft für Public Health)
NGO: Non-governmental organisation
