# Supplementary material for: Priority setting to support a public health research agenda: a modified Delphi study with public health stakeholders in Germany
Source: Health Res Policy Syst. 2023 Aug 28;21:86. doi: 10.1186/s12961-023-01039-w (PMC10463880; doi:10.1186/s12961-023-01039-w)
Supplement: Supplementary file 1 — Additional file 1. List of organisations that were invited to participate in the study—by stakeholder group. We identified specific organisations that fall within each of the included stakeholder groups. The stakeholder groups stand for different professional fields who are either producers, facilitators, or consumers of public health research in Germany. We used this list of organisations for the recruitment of individual respondents for the Delphi stage. [file 12961_2023_1039_MOESM1_ESM.pdf]

Additional file 1: List of organisations that were invited to participate in the study – by stakeholder group

1. Public health research and/or higher education
Bayerischer Forschungs- und Aktionsverbund Public Health e.V.
BfArM – Bundesinstitut für Arzneimittel & Medizin Produkte
BIPS - Leibniz-Institut für Präventionsforschung und Epidemiologie – Bremen
BSPH - Berlin School of Public Health - Alice Salomon Hochschule
BSPH - Berlin School of Public Health - Charité - Universitätsmedizin Berlin
BSPH - Berlin School of Public Health - Technische Universität Berlin
Cochrane Deutschland
Das Helmholtz Zentrum München
DEGAM - Deutsche Gesellschaft für Allgemeinmedizin und Familienmedizin e. V.
DG Pflegewissenschaft e.V.
DGEpi - Deutsche Gesellschaft für Epidemiologie
DGMP - Deutsche Gesellschaft für Medizinische Psychologie e.V.
DGMS - Deutsche Gesellschaft für Medizinische Soziologie e.V.
DGSMP - Deutsche Gesellschaft für Sozialmedizin und Prävention e.V.
DGSP (Deutsche Gesellschaft für Sportmedizin und Prävention)
DIFE - Deutsches Institut für Ernährungsforschung Potsdam-Rehbrücke
DKGW - Deutsche Koordinierungsstelle für Gesundheitswissenschaften
DNVF - Deutsches Netzwerk Versorgungsforschung e.V.
DVGPH - Deutscher Verband für Gesundheitswissenschaften und Public Health e.V.
DVS (Deutsche Vereinigung für Sportwissenschaft)
Freie Universität Berlin - Psychosoziale Prävention und Gesundheitsförderung
GAA - Gesellschaft für Arzneimittelanwendungsforschung und Arzneimittelepidemiologie e. V.
GHUP - Gesellschaft für Hygiene, Umweltmedizin und Prävention
GIZ - Deutsche Gesellschaft für Internationale Zusammenarbeit - Abteilung Gesundheit, Bildung & Soziale Sicherheit
GMDS - Deutsche Gesellschaft für Medizinische Informatik, Biometrie und Epidemiologie e.V.
Hochschule Emden/Leer - Fachbereich Soziale Arbeit und Gesundheit
Hochschule für Angewandte Wissenschaften Hamburg - Public Health - Fakultät Life Sciences
Hochschule für Gesundheit Bochum
Hochschule Furtwangen University - Angewandte Gesundheitswissenschaften

Hochschule Magdeburg-Stendal - Fachgruppe Gesundheitsförderung
Hochschule Neubrandenburg, Gesundheitswissenschaften
IaG - Hochschule Coburg - Institut für angewandte Gesundheitswissenschaften
Ifgs – Institut für Gesundheit & Soziales – die Hochschule für Berufstätige
Ifw – Institut für Fort- und Weiterbildung - Weiterbildungsstudiengang Kommunales Gesundheitsmanagement
IPP - Universität Bremen - Institut für Public Health und Pflegeforschung
ISE - Institut für Sozialmedizin und Epidemiologie - Medizinischen Fakultät Universität zu Lübeck
IZPH - Interdisziplinäres Zentrum für Public Health, Erlangen
Jade-Hochschule - Abt. Technik und Gesundheit für Menschen
Katholischen Hochschule für Sozialwesen Berlin - Instituts für Soziale Gesundheit
LMU - Ludwig-Maximilians-Universität München - Institut f. Med. Informationsverarbeitung, Biometrie & Epidemiologie
Mannheimer Institut für Public Health, Sozial- und Präventivmedizin
Martin-Luther-Universität Halle-Wittenberg - Institut für Gesundheits- und Pflegewissenschaft
Medizinische Hochschule Hannover - Abteilung Epidemiologie, Sozialmedizin und Gesundheitssystemforschung
Norddeutscher Forschungsverbund Public Health
Pädagogische Hochschule Freiburg - Institut für Alltagskultur, Bewegung und Gesundheit
Pädagogische Hochschule Schwäbisch Gmünd - Gesundheitsförderung - Institut für Humanwissenschaften
PEI – Paul-Ehrlich-Institut
PHN - Hochschule Fulda - Public Health and Nutrition
PH Hochschule Fulda - Public Health
RKI - Robert-Koch Institut
TU Chemnitz - Public Health mit Schwerpunkt Prävention und Evaluation
TU Dresden - Medizinische Fakultät - Zentrum für Evidenzbasierte Gesundheitsversorgung (ZEGV)
TU München - Fachrichtung Gesundheits- und Pflegewissenschaft
Universität Bielefeld - Public Health - Health Communication
Universität Düsseldorf - Institut für Medizinische Soziologie
Universität Leipzig - Das Institut für Gesundheitssport und Public Health
Universität Tübingen - Institut für Arbeitsmedizin, Sozialmedizin und Versorgungsforschung
Universitätsklinikum Heidelberg - Institut für Public Health
Universitätsmedizin Greifswald - Institut für Sozialmedizin und Prävention
WIdO - Das Wissenschaftliche Institut der AOK
WINEG - Wissenschaftliches Institut der TK für Nutzen und Effizienz im Gesundheitswesen

ZAG - Zentrum für angewandte Gesundheitswissenschaften der Leuphana Universität Lüneburg
Zi - Zentralinstitut für die kassenärztliche Versorgung
ZVFK - Zentrum für Versorgungsforschung Köln - Universität Köln

2

**2. Public health administration and/or politics**
AOLG - Arbeitsgemeinschaft der Obersten Landesgesundheitsbehörden
BAuA - Bundesanstalt für Arbeitsschutz und Arbeitsmedizin
BfR Bundesinstitut für Risikobewertung
BMAS - Bundesministerium für Arbeit und Soziales
BMG – Bundesministerium für Gesundheit
BMUB - Bundesministerium für Umwelt, Naturschutz, Bau und Reaktorsicherheit
BZgA – Bundeszentrale für gesundheitliche Aufklärung
Der Deutsche Ethikrat
Gesundheitsausschuss des Bundestages
GMK - Gesundheitsministerkonferenz der Länder
ÖGD – Öffentlicher Gesundheitsdienst - Baden-Württemberg
ÖGD – Öffentlicher Gesundheitsdienst - Bayern
ÖGD – Öffentlicher Gesundheitsdienst - Berlin
ÖGD – Öffentlicher Gesundheitsdienst - Brandenburg
ÖGD – Öffentlicher Gesundheitsdienst - Bremen
ÖGD – Öffentlicher Gesundheitsdienst - Hessen
ÖGD – Öffentlicher Gesundheitsdienst - Mecklenburg-Vorpommern
ÖGD – Öffentlicher Gesundheitsdienst - Niedersachsen
ÖGD – Öffentlicher Gesundheitsdienst - Nordrhein-Westfalen
ÖGD – Öffentlicher Gesundheitsdienst - Rheinland-Pfalz
SVR – Sachverständigenrat für Gesundheit
UBA - Umweltbundesamt

3

3. Non-governmental organisations (NGO) and representatives of the general public
Aids-Hilfe
Ärzte ohne Grenzen
ASB - Arbeiter Samariter Bund
AWO - Arbeiterwohlfahrt
BAG Selbsthilfe e.V.
BAGSO - Bundesarbeitsgemeinschaft Senioren Organisation
BVPG - Bundesvereinigung Prävention und Gesundheitsförderung e.V
CARE
DCV - Deutscher Caritas Verband
Deutsche Arbeitsgemeinschaft Selbsthilfegruppen
Deutscher Olympischer Sportbund
Deutsches Rotes Kreuz
DHM - Stiftung Deutsches Hygiene Museum
Diakonisches Werk
DPWV - Deutscher Paritätische Wohlfahrtsverband
DTB - Deutscher Turner-Bund
EPHZ - Europäisches Public Health Zentrum NRW
GIZ - Deutsche Gesellschaft für Internationale Zusammenarbeit - Abteilung Gesundheit, Bildung und Soziale Sicherheit
KGSt - Kommunale Gemeinschaftsstelle für Verwaltungsmanagement
Landesvereinigung für Gesundheit(-förderung) - Berlin-Brandenburg
Landesvereinigung für Gesundheit(-förderung) - Bremen
Landesvereinigung für Gesundheit(-förderung) - Hamburg
Landesvereinigung für Gesundheit(-förderung) - Hessen
Landesvereinigung für Gesundheit(-förderung) - Mecklenburg-Vorpommern
Landesvereinigung für Gesundheit(-förderung) - Niedersachsen
Landesvereinigung für Gesundheit(-förderung) - Rheinland-Pfalz
Landesvereinigung für Gesundheit(-förderung) - Sachsen

Landesvereinigung für Gesundheit(-förderung) - Sachsen-Anhalt
Landesvereinigung für Gesundheit(-förderung) - Schleswig-Holstein
Landesvereinigung für Gesundheit(-förderung) - Thüringen
Sozialverband VdK Deutschland
UPD - Unabhängige Patientenberatung Deutschland
Medico International
World Vision

4

**4. Representatives of health professionals and health care institutions**
Berufsverband Deutscher Psychologinnen und Psychologen - Fachbereich Gesundheitspsychologie
Bundesärztekammer
Bundesverband der Ärzte und Ärztinnen des Öffentlichen Gesundheitsdienstes
Bundeszahnärztekammer
Deutscher Pflegerat
DKG – Deutsche Krankenhausgesellschaft
DNGFK Deutsches Netz gesundheitsfördernder Krankenhäuser
MFT – Medizinischer Fakultätentag
VUD – Verband der Universitätsklinika

5

**5. Self-governing associations of health providers and statutory health insurance**
AOK-Bundesverband
BKK Dachverband
DGUV - Deutsche Gesetzliche Unfallversicherung
DRV - Deutsche Rentenversicherung
G-BA – Gemeinsamer Bundesausschuss
GKV-Spitzenverband
IKK e. V.
KBV – Kassenärztliche Bundesvereinigung

Knappschaft
KZBV - Kassenzahnärztliche Bundesvereinigung
IDZ - Institut der Deutschen Zahnärzte
SVLFG Sozialversicherung für Landwirtschaft, Forsten und Gartenbau
VdEK e.V.
