# Supplementary material for: Priority setting to support a public health research agenda: a modified Delphi study with public health stakeholders in Germany
Source: Health Res Policy Syst. 2023 Aug 28;21:86. doi: 10.1186/s12961-023-01039-w (PMC10463880; doi:10.1186/s12961-023-01039-w)
Supplement: Supplementary file 2 — Additional file 2. Final list of research topics after content analysis—Incl. how many respondents´ suggestions were aggregated into the research topic. This file represents the results of the content analysis after the first Delphi round. In total, the respondents proposed 529 research topics and 50 assessment criteria in the first Delphi round, which we aggregated through a content analysis into 76 sufficiently distinct research topics and 6 assessment criteria, respectively. a and 2b show the final list of research topics based on the content analysis for the substantive and the methodological-theoretical research topics, respectively. [file 12961_2023_1039_MOESM2_ESM.pdf]

Additional file 2a: Final list of substantive research topics after content analysis - Incl. how many respondents´ suggestions were aggregated into the research topic

	Research topic	***Nr. of Suggestions***
1	Health in all policies	22
2	Interventions in settings / settings-changing measures	21
3	Digitisation and health	19
4	Governance (global, national, regional) and health systems	18
5	Community-based prevention and health promotion	17
6	Social inequality and injustice	17
7	Diversity and gender	16
8	Health literacy promotion	15
9	Research with focus on specific target groups	14
10	Knowledge translation	14
11	Improving health care	14
12	Influence through economisation and interest groups	13
13	Research on health professions	13
14	Environment/climate change and health	13
15	Behaviour change measures	13
16	Impact of health policy measures	11
17	Implementation research	11
18	Health and children/youth/family	10
19	Global Health and effects of globalisation	10
20	Patient and user orientation	10
21	Work and health	9
22	Life course perspective	8
23	Mental health	8
24	Public health crises and disasters	8
25	Population perspective on pharmaceuticals	7
26	Infectious diseases and vaccination protection	7
27	Migration health	7
28	Prevention of non-communicable diseases	7

29	Disability and multimorbidity	6
30	Nutrition and health (cultural, physiological, social)	6
31	One Health	6
32	Resilience	6
33	Self-help	6
34	Sustainable Development Goals (SDGs)	6
35	Effectiveness of municipal / community-oriented approaches	6
36	Health policy analysis	5
37	Health and ageing	5
38	Health communication	5
39	Health economic evaluation	5
40	Sustainability	5
41	Interdependencies between society, setting and individual health	5
42	Effectiveness of counselling at the individual level	5
43	Research on health care needs	4
44	Health reporting	4
45	Intervention studies over longer periods of time	4
46	Accidents, violence, self-harm	4

Additional file 2b: Final list of methodological-theoretical topics after content analysis - Incl. how many respondents´ suggestions were aggregated into the research topic

	Research topic	*Nr. of Suggestions*
1	Participation in health research	23
2	Sociological aspects of health	15
3	Evidence-based public health research	13
4	Population participation	9
5	Methodological research on registry and routine data	8
6	Big Data	7
7	Indicators for the quality of health care and public health	7
8	Further development of intervention studies	7
9	Causal analyses / experiments	6
10	Transdisciplinary research	6
11	Interdisciplinary research	5
12	Systematic reviews	5
13	Indicators of quality of life and of positive health states	4
14	Complex interventions	4
15	Modelling studies - Decision Analysis	4
16	Research on public health theories	3
17	Conceptualisation of behaviour/relationship	3
18	Qualitative health studies	3
19	Structural and process indicators	3
20	Development and maintenance of good health	2
21	Indicators for health targets	2
22	Indicators of health literacy	2
23	Internationally comparable indicators	2
24	Mobility concepts	2
25	Online Social Research	2
26	Process evaluation	2

27	Action research	1
28	Comparative Effectiveness Research (CER)	1
29	Preventive marker	1
30	Theoretical foundation of effects models	1
