# Supplementary material for: Priority setting to support a public health research agenda: a modified Delphi study with public health stakeholders in Germany
Source: Health Res Policy Syst. 2023 Aug 28;21:86. doi: 10.1186/s12961-023-01039-w (PMC10463880; doi:10.1186/s12961-023-01039-w)
Supplement: Supplementary file 3 — Additional file 3. Comparison of the ranking of research topics between public health researchers versus public health practitioners. a and b show the comparison of the ranking of substantive and methodological-theoretical research topics, respectively, by stakeholder group (public health researchers versus public health practitioners). A difference in minus means the research topic is ranked higher by public health researchers; a difference in plus means the research topic is ranked higher by public health practitioners. [file 12961_2023_1039_MOESM3_ESM.pdf]

**Additional file 3a**: Comparison of the ranking of substantive research topics between public health researchers versus public health practitioners – all three criteria for substantive research topics combined

*(A difference in minus means the research topic is ranked higher by public health researchers; a difference in plus means the research topic is ranked higher by public health practitioners).*

| Research topic | Rank PH research | Rank PH practice | *Difference in ranking* | *Difference in actual score* |
|---|---|---|---|---|
| Interventions in settings / setting-changing measures | 1 | 1 | *0* | *-0.24* |
| Health in all policies | 2 | 2 | *0* | *-0.02* |
| Social inequality and injustice | 3 | 4 | *-1* | *-0.05* |
| Community-based prevention and health promotion | 4 | 6 | *-2* | *-0.09* |
| Implementation research | 5 | 5 | *0* | *-0.05* |
| Impact of health policy measures | 6 | 3 | *3* | *0.01* |
| Improving health care | 7 | 11 | *-4* | *-0.17* |
| Intervention studies over longer periods of time | 8 | 9 | *-1* | *-0.07* |
| Effectiveness of municipal / community-oriented approaches | 9 | 10 | *-1* | *-0.03* |
| Health and children/youth/family | 10 | 18 | *-8* | *-0.15* |
| Research with focus on specific target groups | 11 | 23 | *-12* | *-0.22* |
| Health literacy promotion | 12 | 7 | *5* | *0.04* |
| Interdependencies between society, setting and individual health | 13 | 14 | *-1* | *-0.04* |
| Health and ageing | 14 | 12 | *2* | *0.00* |
| Knowledge translation | 15 | 19 | *-4* | *-0.07* |
| Patient and user orientation | 16 | 22 | *-6* | *-0.10* |
| Environment/climate change and health | 17 | 20 | *-3* | *-0.04* |
| Work and health | 18 | 28 | *-10* | *-0.21* |
| Global health and effects of globalisation | 19 | 13 | *6* | *0.07* |
| Governance (global, national, regional) and health systems | 20 | 33 | *-13* | *-0.27* |
| Mental health | 21 | 30 | *-9* | *-0.20* |
| Digitisation and health | 22 | 8 | *14* | *0.18* |
| Disability and multimorbidity | 23 | 34 | *-11* | *-0.26* |
| Life course perspective | 24 | 27 | *-3* | *-0.13* |
| Research on health care needs | 25 | 24 | *1* | *-0.03* |
| Influence through economisation and interest groups | 26 | 17 | *9* | *0.10* |

| | | | | |
|---|---|---|---|---|
| Prevention of non-communicable diseases | 27 | 16 | *11* | *0.13* |
| Health communication | 28 | 15 | *13* | *0.16* |
| Migration health | 29 | 25 | *4* | *0.02* |
| Sustainability | 30 | 32 | *-2* | *-0.10* |
| Health policy analysis | 31 | 26 | *5* | *0.00* |
| Sustainable Development Goals (SDGs) | 32 | 38 | *-6* | *-0.12* |
| Behaviour change measures | 33 | 40 | *-7* | *-0.14* |
| Resilience | 34 | 35 | *-1* | *-0.07* |
| Nutrition and health (cultural, physiological, social) | 35 | 21 | *14* | *0.18* |
| Diversity and gender | 36 | 31 | *5* | *0.06* |
| Self-help | 37 | 45 | *-8* | *-0.22* |
| Research on health professions | 38 | 36 | *2* | *0.07* |
| Health reporting | 39 | 37 | *2* | *0.10* |
| One Health | 40 | 39 | *1* | *0.08* |
| Effectiveness of counselling at the individual level | 41 | 42 | *-1* | *-0.09* |
| Health economic evaluation | 42 | 43 | *-1* | *-0.01* |
| Infectious diseases and vaccination protection | 43 | 29 | *14* | *0.30* |
| Accidents, violence, self-harm | 44 | 46 | *-2* | *-0.13* |
| Population perspective on pharmaceuticals | 45 | 41 | *4* | *0.13* |
| Public health crises and disasters | 46 | 44 | *2* | *0.07* |
| **Average** | | | ***0.00*** | ***-0.04*** |

6

**Additional file 3b**: Comparison of the ranking of methodological-theoretical research topics between public health researchers versus public health practitioners – all three criteria combined

*(A difference in minus means the research topic is ranked higher by public health researchers; a difference in plus means the research topic is ranked higher by public health practitioners).*

| **Research topic** | **Rank PH research** | **Rank PH practice** | ***Difference in ranking*** | ***Difference in actual score*** |
|---|---|---|---|---|
| Complex interventions | 1 | 3 | *-2* | *-0.23* |
| Interdisciplinary research | 2 | 1 | *1* | *-0.15* |
| Evidence-based public health research | 3 | 4 | *-1* | *-0.14* |
| Transdisciplinary research | 4 | 10 | *-6* | *-0.30* |
| Further development of intervention studies | 5 | 5 | *0* | *-0.19* |
| Participation in health research | 6 | 6 | *0* | *-0.10* |
| Structural and process indicators | 7 | 18 | *-11* | *-0.33* |
| Causal analyses / experiments | 8 | 22 | *-14* | *-0.34* |
| Process evaluation | 9 | 24 | *-15* | *-0.32* |
| Systematic reviews | 10 | 12 | *-2* | *-0.12* |
| Population participation | 11 | 7 | *4* | *0.05* |
| Indicators for the quality of health care and public health | 12 | 2 | *10* | *0.24* |
| Indicators of quality of life and of positive health states | 13 | 19 | *-6* | *-0.24* |
| Indicators of health literacy | 14 | 13 | *1* | *-0.08* |
| Conceptualization of behaviour/relationship | 15 | 23 | *-8* | *-0.26* |
| Development and maintenance of good health | 16 | 8 | *8* | *0.09* |
| Methodological research on registry and routine data | 17 | 16 | *1* | *-0.11* |
| Sociological aspects of health | 18 | 20 | *-2* | *-0.16* |
| Comparative Effectiveness Research (CER) | 19 | 17 | *2* | *-0.13* |
| Theoretical foundation of effects models | 20 | 28 | *-8* | *-0.37* |
| Internationally comparable indicators | 21 | 9 | *12* | *0.12* |
| Qualitative health studies | 22 | 11 | *11* | *0.06* |
| Research on public health theories | 23 | 29 | *-6* | *-0.38* |
| Big Data | 24 | 15 | *9* | *0.07* |

| | | | | |
|---|---|---|---|---|
| Action research | 25 | 26 | *-1* | *-0.05* |
| Indicators for health targets | 26 | 25 | *1* | *-0.03* |
| Preventive markers | 27 | 27 | *0* | *-0.01* |
| Modelling studies - Decision Analysis | 28 | 14 | *14* | *0.26* |
| Mobility concepts | 29 | 30 | *-1* | *-0.16* |
| Online Social Research | 30 | 21 | *9* | *0.28* |
| **Average** | | | ***0.00*** | ***-0.10*** |

12
